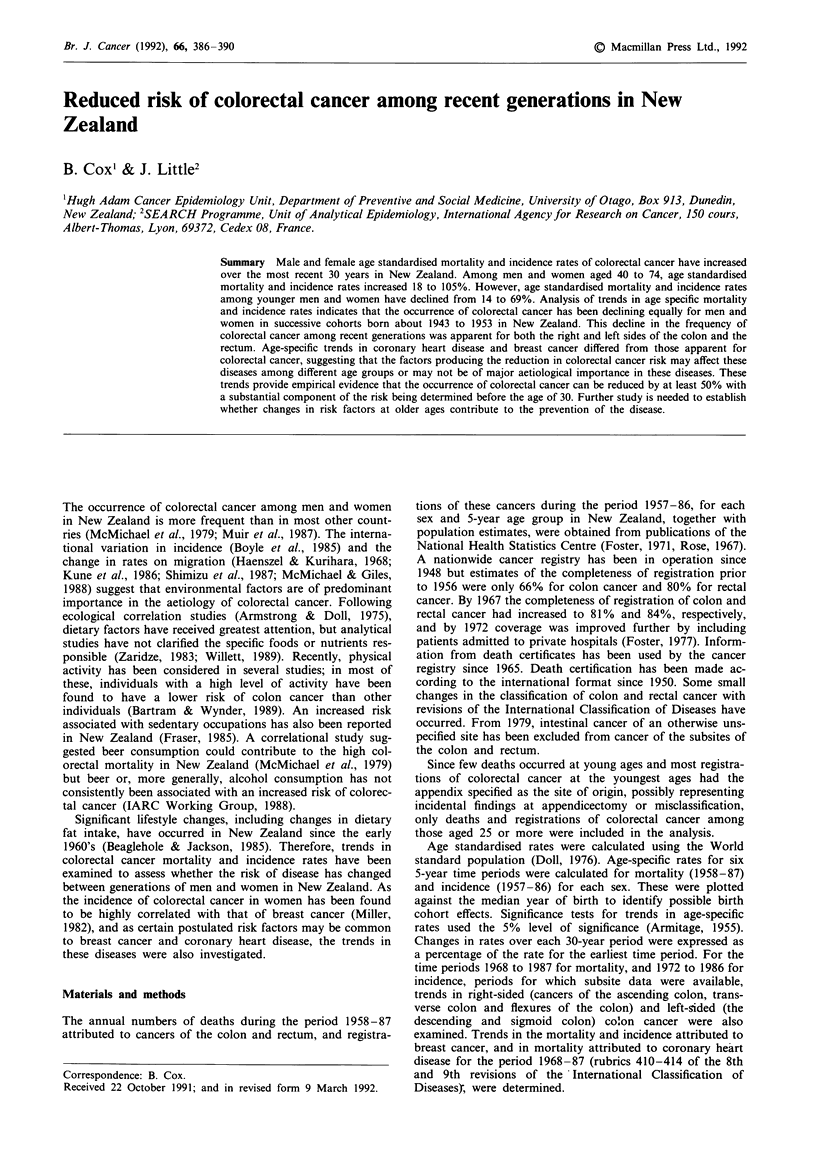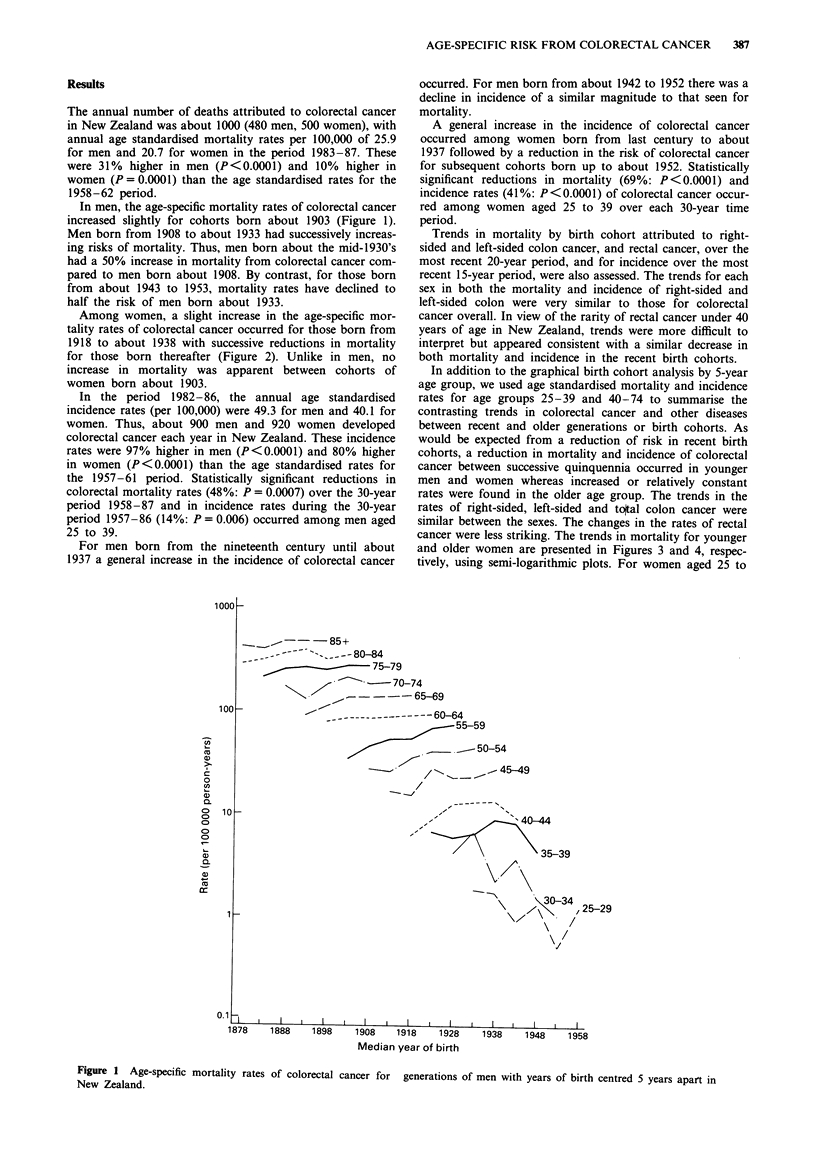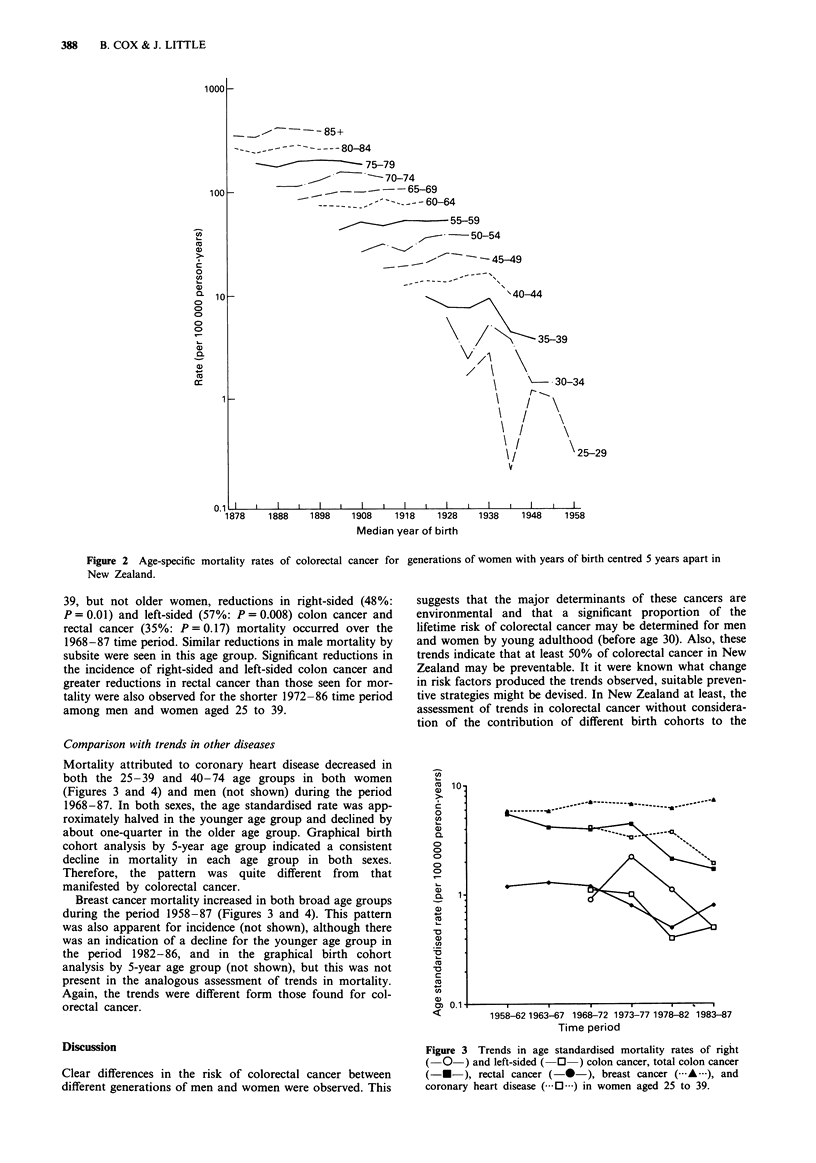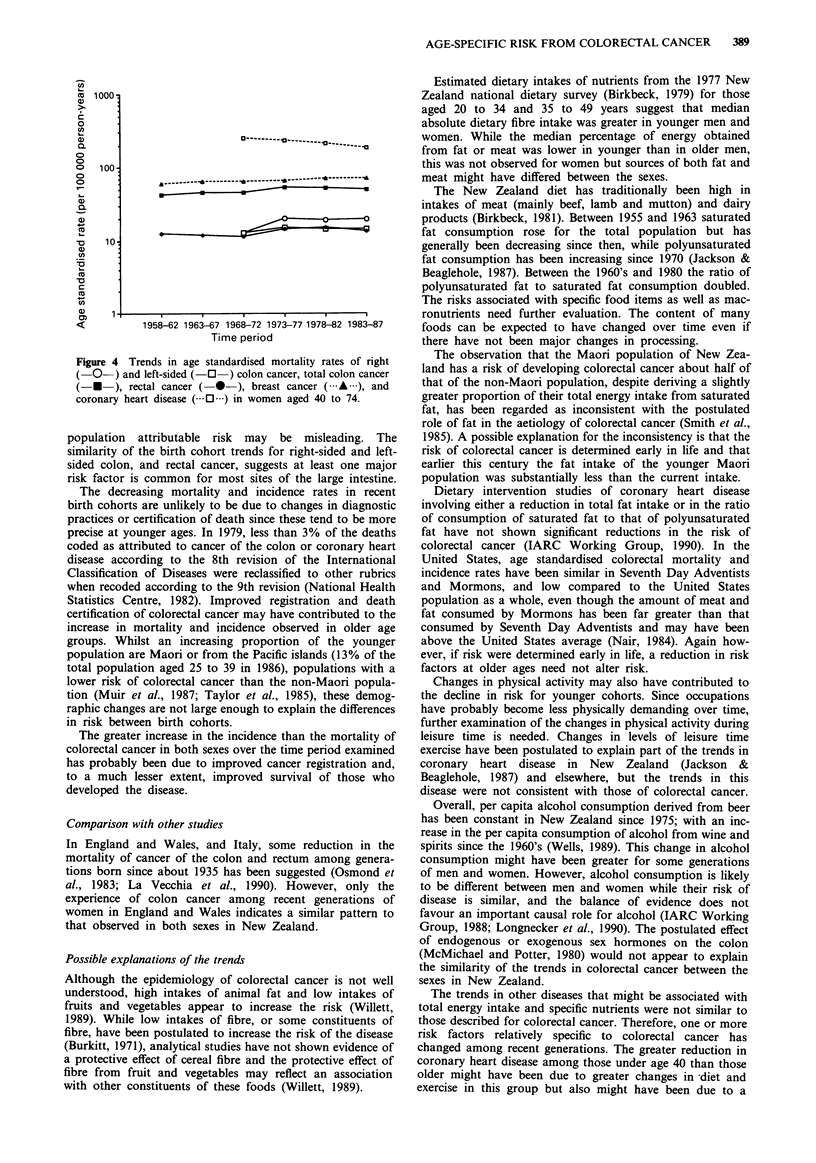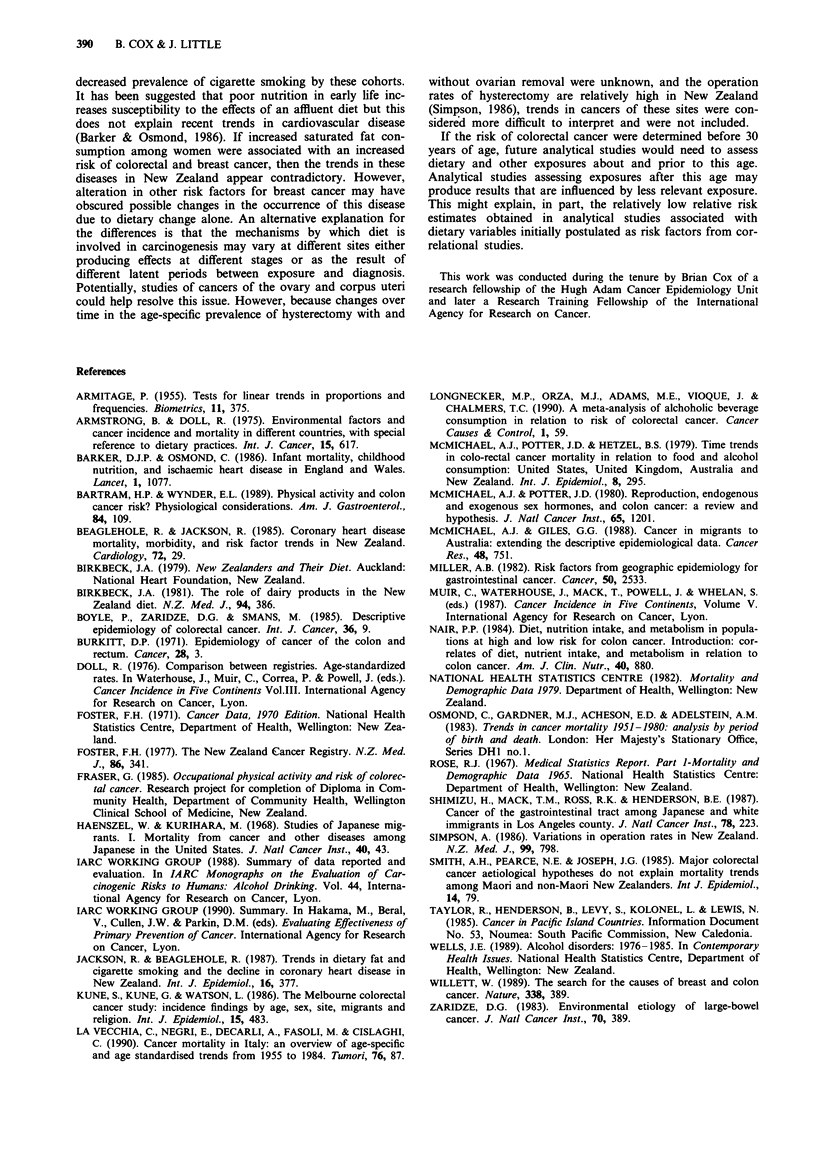# Reduced risk of colorectal cancer among recent generations in New Zealand.

**DOI:** 10.1038/bjc.1992.274

**Published:** 1992-08

**Authors:** B. Cox, J. Little

**Affiliations:** Hugh Adam Cancer Epidemiology Unit, Department of Preventive and Social Medicine, University of Otago, Dunedin, New Zealand.

## Abstract

Male and female age standardised mortality and incidence rates of colorectal cancer have increased over the most recent 30 years in New Zealand. Among men and women aged 40 to 74, age standardised mortality and incidence rates increased 18 to 105%. However, age standardised mortality and incidence rates among younger men and women have declined from 14 to 69%. Analysis of trends in age specific mortality and incidence rates indicates that the occurrence of colorectal cancer has been declining equally for men and women in successive cohorts born about 1943 to 1953 in New Zealand. This decline in the frequency of colorectal cancer among recent generations was apparent for both the right and left sides of the colon and the rectum. Age-specific trends in coronary heart disease and breast cancer differed from those apparent for colorectal cancer, suggesting that the factors producing the reduction in colorectal cancer risk may affect these diseases among different age groups or may not be of major aetiological importance in these diseases. These trends provide empirical evidence that the occurrence of colorectal cancer can be reduced by at least 50% with a substantial component of the risk being determined before the age of 30. Further study is needed to establish whether changes in risk factors at older ages contribute to the prevention of the disease.


Br. J. Cancer (1992), 66, 386–390 © Macmillan Press Ltd., 1992

### Reduced risk of colorectal cancer among recent generations in New Zealand

B. Cox[1] & J. Little[2]

[1]*Hugh Adam Cancer Epidemiology Unit, Department of Preventive and Social Medicine, University of Otago, Box 913, Dunedin, New Zealand;* [2]*SEARCH Programme, Unit of Analytical Epidemiology, International Agency for Research on Cancer, 150 cours, Albert-Thomas, Lyon, 69372, Cedex 08, France.*

**Summary** Male and female age standardised mortality and incidence rates of colorectal cancer have increased over the most recent 30 years in New Zealand. Among men and women aged 40 to 74, age standardised mortality and incidence rates increased 18 to 105%. However, age standardised mortality and incidence rates among younger men and women have declined from 14 to 69%. Analysis of trends in age specific mortality and incidence rates indicates that the occurrence of colorectal cancer has been declining equally for men and women in successive cohorts born about 1943 to 1953 in New Zealand. This decline in the frequency of colorectal cancer among recent generations was apparent for both the right and left sides of the colon and the rectum. Age-specific trends in coronary heart disease and breast cancer differed from those apparent for colorectal cancer, suggesting that the factors producing the reduction in colorectal cancer risk may affect these diseases among different age groups or may not be of major aetiological importance in these diseases. These trends provide empirical evidence that the occurrence of colorectal cancer can be reduced by at least 50% with a substantial component of the risk being determined before the age of 30. Further study is needed to establish whether changes in risk factors at older ages contribute to the prevention of the disease.

The occurrence of colorectal cancer among men and women in New Zealand is more frequent than in most other countries (McMichael *et al.*, 1979; Muir *et al.*, 1987). The international variation in incidence (Boyle *et al.*, 1985) and the change in rates on migration (Haenszel & Kurihara, 1968; Kune *et al.*, 1986; Shimizu *et al.*, 1987; McMichael & Giles, 1988) suggest that environmental factors are of predominant importance in the aetiology of colorectal cancer. Following ecological correlation studies (Armstrong & Doll, 1975), dietary factors have received greatest attention, but analytical studies have not clarified the specific foods or nutrients responsible (Zaridze, 1983; Willett, 1989). Recently, physical activity has been considered in several studies; in most of these, individuals with a high level of activity have been found to have a lower risk of colon cancer than other individuals (Bartram & Wynder, 1989). An increased risk associated with sedentary occupations has also been reported in New Zealand (Fraser, 1985). A correlational study suggested beer consumption could contribute to the high colorectal mortality in New Zealand (McMichael *et al.*, 1979) but beer or, more generally, alcohol consumption has not consistently been associated with an increased risk of colorectal cancer (IARC Working Group, 1988).

Significant lifestyle changes, including changes in dietary fat intake, have occurred in New Zealand since the early 1960's (Beaglehole & Jackson, 1985). Therefore, trends in colorectal cancer mortality and incidence rates have been examined to assess whether the risk of disease has changed between generations of men and women in New Zealand. As the incidence of colorectal cancer in women has been found to be highly correlated with that of breast cancer (Miller, 1982), and as certain postulated risk factors may be common to breast cancer and coronary heart disease, the trends in these diseases were also investigated.

### Materials and methods

The annual numbers of deaths during the period 1958–87 attributed to cancers of the colon and rectum, and registrations of these cancers during the period 1957–86, for each sex and 5-year age group in New Zealand, together with population estimates, were obtained from publications of the National Health Statistics Centre (Foster, 1971, Rose, 1967). A nationwide cancer registry has been in operation since 1948 but estimates of the completeness of registration prior to 1956 were only 66% for colon cancer and 80% for rectal cancer. By 1967 the completeness of registration of colon and rectal cancer had increased to 81% and 84%, respectively, and by 1972 coverage was improved further by including patients admitted to private hospitals (Foster, 1977). Information from death certificates has been used by the cancer registry since 1965. Death certification has been made according to the international format since 1950. Some small changes in the classification of colon and rectal cancer with revisions of the International Classification of Diseases have occurred. From 1979, intestinal cancer of an otherwise unspecified site has been excluded from cancer of the subsites of the colon and rectum.

Since few deaths occurred at young ages and most registrations of colorectal cancer at the youngest ages had the appendix specified as the site of origin, possibly representing incidental findings at appendicectomy or misclassification, only deaths and registrations of colorectal cancer among those aged 25 or more were included in the analysis.

Age standardised rates were calculated using the World standard population (Doll, 1976). Age-specific rates for six 5-year time periods were calculated for mortality (1958–87) and incidence (1957–86) for each sex. These were plotted against the median year of birth to identify possible birth cohort effects. Significance tests for trends in age-specific rates used the 5% level of significance (Armitage, 1955). Changes in rates over each 30-year period were expressed as a percentage of the rate for the earliest time period. For the time periods 1968 to 1987 for mortality, and 1972 to 1986 for incidence, periods for which subsite data were available, trends in right-sided (cancers of the ascending colon, transverse colon and flexures of the colon) and left-sided (the descending and sigmoid colon) colon cancer were also examined. Trends in the mortality and incidence attributed to breast cancer, and in mortality attributed to coronary heart disease for the period 1968–87 (rubrics 410–414 of the 8th and 9th revisions of the International Classification of Diseases), were determined.

Correspondence: B. Cox.
Received 22 October 1991; and in revised form 9 March 1992.

**Results**

The annual number of deaths attributed to colorectal cancer in New Zealand was about 1000 (480 men, 500 women), with annual age standardised mortality rates per 100,000 of 25.9 for men and 20.7 for women in the period 1983–87. These were 31% higher in men ($P<0.0001$) and 10% higher in women ($P=0.0001$) than the age standardised rates for the 1958–62 period.

In men, the age-specific mortality rates of colorectal cancer increased slightly for cohorts born about 1903 (Figure 1). Men born from 1908 to about 1933 had successively increasing risks of mortality. Thus, men born about the mid-1930's had a 50% increase in mortality from colorectal cancer compared to men born about 1908. By contrast, for those born from about 1943 to 1953, mortality rates have declined to half the risk of men born about 1933.

Among women, a slight increase in the age-specific mortality rates of colorectal cancer occurred for those born from 1918 to about 1938 with successive reductions in mortality for those born thereafter (Figure 2). Unlike in men, no increase in mortality was apparent between cohorts of women born about 1903.

In the period 1982–86, the annual age standardised incidence rates (per 100,000) were 49.3 for men and 40.1 for women. Thus, about 900 men and 920 women developed colorectal cancer each year in New Zealand. These incidence rates were 97% higher in men ($P<0.0001$) and 80% higher in women ($P<0.0001$) than the age standardised rates for the 1957–61 period. Statistically significant reductions in colorectal mortality rates (48%: $P=0.0007$) over the 30-year period 1958–87 and in incidence rates during the 30-year period 1957–86 (14%: $P=0.006$) occurred among men aged 25 to 39.

For men born from the nineteenth century until about 1937 a general increase in the incidence of colorectal cancer occurred. For men born from about 1942 to 1952 there was a decline in incidence of a similar magnitude to that seen for mortality.

A general increase in the incidence of colorectal cancer occurred among women born from last century to about 1937 followed by a reduction in the risk of colorectal cancer for subsequent cohorts born up to about 1952. Statistically significant reductions in mortality (69%: $P<0.0001$) and incidence rates (41%: $P<0.0001$) of colorectal cancer occurred among women aged 25 to 39 over each 30-year time period.

Trends in mortality by birth cohort attributed to right-sided and left-sided colon cancer, and rectal cancer, over the most recent 20-year period, and for incidence over the most recent 15-year period, were also assessed. The trends for each sex in both the mortality and incidence of right-sided and left-sided colon were very similar to those for colorectal cancer overall. In view of the rarity of rectal cancer under 40 years of age in New Zealand, trends were more difficult to interpret but appeared consistent with a similar decrease in both mortality and incidence in the recent birth cohorts.

In addition to the graphical birth cohort analysis by 5-year age group, we used age standardised mortality and incidence rates for age groups 25–39 and 40–74 to summarise the contrasting trends in colorectal cancer and other diseases between recent and older generations or birth cohorts. As would be expected from a reduction of risk in recent birth cohorts, a reduction in mortality and incidence of colorectal cancer between successive quinquennia occurred in younger men and women whereas increased or relatively constant rates were found in the older age group. The trends in the rates of right-sided, left-sided and total colon cancer were similar between the sexes. The changes in the rates of rectal cancer were less striking. The trends in mortality for younger and older women are presented in Figures 3 and 4, respectively, using semi-logarithmic plots. For women aged 25 to

**Figure 1** Age-specific mortality rates of colorectal cancer for generations of men with years of birth centred 5 years apart in New Zealand.

**Figure 2** Age-specific mortality rates of colorectal cancer for generations of women with years of birth centred 5 years apart in New Zealand.

39, but not older women, reductions in right-sided (48%: $P = 0.01$) and left-sided (57%: $P = 0.008$) colon cancer and rectal cancer (35%: $P = 0.17$) mortality occurred over the 1968–87 time period. Similar reductions in male mortality by subsite were seen in this age group. Significant reductions in the incidence of right-sided and left-sided colon cancer and greater reductions in rectal cancer than those seen for mortality were also observed for the shorter 1972–86 time period among men and women aged 25 to 39.

*Comparison with trends in other diseases*

Mortality attributed to coronary heart disease decreased in both the 25–39 and 40–74 age groups in both women (Figures 3 and 4) and men (not shown) during the period 1968–87. In both sexes, the age standardised rate was approximately halved in the younger age group and declined by about one-quarter in the older age group. Graphical birth cohort analysis by 5-year age group indicated a consistent decline in mortality in each age group in both sexes. Therefore, the pattern was quite different from that manifested by colorectal cancer.

Breast cancer mortality increased in both broad age groups during the period 1958–87 (Figures 3 and 4). This pattern was also apparent for incidence (not shown), although there was an indication of a decline for the younger age group in the period 1982–86, and in the graphical birth cohort analysis by 5-year age group (not shown), but this was not present in the analogous assessment of trends in mortality. Again, the trends were different form those found for colorectal cancer.

### Discussion

Clear differences in the risk of colorectal cancer between different generations of men and women were observed. This suggests that the major determinants of these cancers are environmental and that a significant proportion of the lifetime risk of colorectal cancer may be determined for men and women by young adulthood (before age 30). Also, these trends indicate that at least 50% of colorectal cancer in New Zealand may be preventable. It it were known what change in risk factors produced the trends observed, suitable preventive strategies might be devised. In New Zealand at least, the assessment of trends in colorectal cancer without consideration of the contribution of different birth cohorts to the

**Figure 3** Trends in age standardised mortality rates of right (—O—) and left-sided (—□—) colon cancer, total colon cancer (—■—), rectal cancer (—●—), breast cancer (···▲···), and coronary heart disease (···□···) in women aged 25 to 39.

**Figure 4** Trends in age standardised mortality rates of right (—O—) and left-sided (—□—) colon cancer, total colon cancer (—■—), rectal cancer (—●—), breast cancer (···▲···), and coronary heart disease (···□···) in women aged 40 to 74.

population attributable risk may be misleading. The similarity of the birth cohort trends for right-sided and left-sided colon, and rectal cancer, suggests at least one major risk factor is common for most sites of the large intestine.

The decreasing mortality and incidence rates in recent birth cohorts are unlikely to be due to changes in diagnostic practices or certification of death since these tend to be more precise at younger ages. In 1979, less than 3% of the deaths coded as attributed to cancer of the colon or coronary heart disease according to the 8th revision of the International Classification of Diseases were reclassified to other rubrics when recoded according to the 9th revision (National Health Statistics Centre, 1982). Improved registration and death certification of colorectal cancer may have contributed to the increase in mortality and incidence observed in older age groups. Whilst an increasing proportion of the younger population are Maori or from the Pacific islands (13% of the total population aged 25 to 39 in 1986), populations with a lower risk of colorectal cancer than the non-Maori population (Muir *et al.*, 1987; Taylor *et al.*, 1985), these demographic changes are not large enough to explain the differences in risk between birth cohorts.

The greater increase in the incidence than the mortality of colorectal cancer in both sexes over the time period examined has probably been due to improved cancer registration and, to a much lesser extent, improved survival of those who developed the disease.

*Comparison with other studies*

In England and Wales, and Italy, some reduction in the mortality of cancer of the colon and rectum among generations born since about 1935 has been suggested (Osmond *et al.*, 1983; La Vecchia *et al.*, 1990). However, only the experience of colon cancer among recent generations of women in England and Wales indicates a similar pattern to that observed in both sexes in New Zealand.

*Possible explanations of the trends*

Although the epidemiology of colorectal cancer is not well understood, high intakes of animal fat and low intakes of fruits and vegetables appear to increase the risk (Willett, 1989). While low intakes of fibre, or some constituents of fibre, have been postulated to increase the risk of the disease (Burkitt, 1971), analytical studies have not shown evidence of a protective effect of cereal fibre and the protective effect of fibre from fruit and vegetables may reflect an association with other constituents of these foods (Willett, 1989).

Estimated dietary intakes of nutrients from the 1977 New Zealand national dietary survey (Birkbeck, 1979) for those aged 20 to 34 and 35 to 49 years suggest that median absolute dietary fibre intake was greater in younger men and women. While the median percentage of energy obtained from fat or meat was lower in younger than in older men, this was not observed for women but sources of both fat and meat might have differed between the sexes.

The New Zealand diet has traditionally been high in intakes of meat (mainly beef, lamb and mutton) and dairy products (Birkbeck, 1981). Between 1955 and 1963 saturated fat consumption rose for the total population but has generally been decreasing since then, while polyunsaturated fat consumption has been increasing since 1970 (Jackson & Beaglehole, 1987). Between the 1960's and 1980 the ratio of polyunsaturated fat to saturated fat consumption doubled. The risks associated with specific food items as well as macronutrients need further evaluation. The content of many foods can be expected to have changed over time even if there have not been major changes in processing.

The observation that the Maori population of New Zealand has a risk of developing colorectal cancer about half of that of the non-Maori population, despite deriving a slightly greater proportion of their total energy intake from saturated fat, has been regarded as inconsistent with the postulated role of fat in the aetiology of colorectal cancer (Smith *et al.*, 1985). A possible explanation for the inconsistency is that the risk of colorectal cancer is determined early in life and that earlier this century the fat intake of the younger Maori population was substantially less than the current intake.

Dietary intervention studies of coronary heart disease involving either a reduction in total fat intake or in the ratio of consumption of saturated fat to that of polyunsaturated fat have not shown significant reductions in the risk of colorectal cancer (IARC Working Group, 1990). In the United States, age standardised colorectal mortality and incidence rates have been similar in Seventh Day Adventists and Mormons, and low compared to the United States population as a whole, even though the amount of meat and fat consumed by Mormons has been far greater than that consumed by Seventh Day Adventists and may have been above the United States average (Nair, 1984). Again however, if risk were determined early in life, a reduction in risk factors at older ages need not alter risk.

Changes in physical activity may also have contributed to the decline in risk for younger cohorts. Since occupations have probably become less physically demanding over time, further examination of the changes in physical activity during leisure time is needed. Changes in levels of leisure time exercise have been postulated to explain part of the trends in coronary heart disease in New Zealand (Jackson & Beaglehole, 1987) and elsewhere, but the trends in this disease were not consistent with those of colorectal cancer.

Overall, per capita alcohol consumption derived from beer has been constant in New Zealand since 1975; with an increase in the per capita consumption of alcohol from wine and spirits since the 1960's (Wells, 1989). This change in alcohol consumption might have been greater for some generations of men and women. However, alcohol consumption is likely to be different between men and women while their risk of disease is similar, and the balance of evidence does not favour an important causal role for alcohol (IARC Working Group, 1988; Longnecker *et al.*, 1990). The postulated effect of endogenous or exogenous sex hormones on the colon (McMichael and Potter, 1980) would not appear to explain the similarity of the trends in colorectal cancer between the sexes in New Zealand.

The trends in other diseases that might be associated with total energy intake and specific nutrients were not similar to those described for colorectal cancer. Therefore, one or more risk factors relatively specific to colorectal cancer has changed among recent generations. The greater reduction in coronary heart disease among those under age 40 than those older might have been due to greater changes in diet and exercise in this group but also might have been due to a decreased prevalence of cigarette smoking by these cohorts. It has been suggested that poor nutrition in early life increases susceptibility to the effects of an affluent diet but this does not explain recent trends in cardiovascular disease (Barker & Osmond, 1986). If increased saturated fat consumption among women were associated with an increased risk of colorectal and breast cancer, then the trends in these diseases in New Zealand appear contradictory. However, alteration in other risk factors for breast cancer may have obscured possible changes in the occurrence of this disease due to dietary change alone. An alternative explanation for the differences is that the mechanisms by which diet is involved in carcinogenesis may vary at different sites either producing effects at different stages or as the result of different latent periods between exposure and diagnosis. Potentially, studies of cancers of the ovary and corpus uteri could help resolve this issue. However, because changes over time in the age-specific prevalence of hysterectomy with and without ovarian removal were unknown, and the operation rates of hysterectomy are relatively high in New Zealand (Simpson, 1986), trends in cancers of these sites were considered more difficult to interpret and were not included.

If the risk of colorectal cancer were determined before 30 years of age, future analytical studies would need to assess dietary and other exposures about and prior to this age. Analytical studies assessing exposures after this age may produce results that are influenced by less relevant exposure. This might explain, in part, the relatively low relative risk estimates obtained in analytical studies associated with dietary variables initially postulated as risk factors from correlational studies.

This work was conducted during the tenure by Brian Cox of a research fellowship of the Hugh Adam Cancer Epidemiology Unit and later a Research Training Fellowship of the International Agency for Research on Cancer.

### References

ARMITAGE, P. (1955). Tests for linear trends in proportions and frequencies. *Biometrics*, **11**, 375.

ARMSTRONG, B. & DOLL, R. (1975). Environmental factors and cancer incidence and mortality in different countries, with special reference to dietary practices. *Int. J. Cancer*, **15**, 617.

BARKER, D.J.P. & OSMOND, C. (1986). Infant mortality, childhood nutrition, and ischaemic heart disease in England and Wales. *Lancet*, **1**, 1077.

BARTRAM, H.P. & WYNDER, E.L. (1989). Physical activity and colon cancer risk? Physiological considerations. *Am. J. Gastroenterol.*, **84**, 109.

BEAGLEHOLE, R. & JACKSON, R. (1985). Coronary heart disease mortality, morbidity, and risk factor trends in New Zealand. *Cardiology*, **72**, 29.

BIRKBECK, J.A. (1979). *New Zealanders and Their Diet*. Auckland: National Heart Foundation, New Zealand.

BIRKBECK, J.A. (1981). The role of dairy products in the New Zealand diet. *N.Z. Med. J.*, **94**, 386.

BOYLE, P., ZARIDZE, D.G. & SMANS, M. (1985). Descriptive epidemiology of colorectal cancer. *Int. J. Cancer*, **36**, 9.

BURKITT, D.P. (1971). Epidemiology of cancer of the colon and rectum. *Cancer*, **28**, 3.

DOLL, R. (1976). Comparison between registries. Age-standardized rates. In Waterhouse, J., Muir, C., Correa, P. & Powell, J. (eds.). *Cancer Incidence in Five Continents* Vol.III. International Agency for Research on Cancer, Lyon.

FOSTER, F.H. (1971). *Cancer Data, 1970 Edition*. National Health Statistics Centre, Department of Health, Wellington: New Zealand.

FOSTER, F.H. (1977). The New Zealand Cancer Registry. *N.Z. Med. J.*, **86**, 341.

FRASER, G. (1985). *Occupational physical activity and risk of colorectal cancer*. Research project for completion of Diploma in Community Health, Department of Community Health, Wellington Clinical School of Medicine, New Zealand.

HAENSZEL, W. & KURIHARA, M. (1968). Studies of Japanese migrants. I. Mortality from cancer and other diseases among Japanese in the United States. *J. Natl Cancer Inst.*, **40**, 43.

IARC WORKING GROUP (1988). Summary of data reported and evaluation. In *IARC Monographs on the Evaluation of Carcinogenic Risks to Humans: Alcohol Drinking*. Vol. 44, International Agency for Research on Cancer, Lyon.

IARC WORKING GROUP (1990). Summary. In Hakama, M., Beral, V., Cullen, J.W. & Parkin, D.M. (eds). *Evaluating Effectiveness of Primary Prevention of Cancer*. International Agency for Research on Cancer, Lyon.

JACKSON, R. & BEAGLEHOLE, R. (1987). Trends in dietary fat and cigarette smoking and the decline in coronary heart disease in New Zealand. *Int. J. Epidemiol.*, **16**, 377.

KUNE, S., KUNE, G. & WATSON, L. (1986). The Melbourne colorectal cancer study: incidence findings by age, sex, site, migrants and religion. *Int. J. Epidemiol.*, **15**, 483.

LA VECCHIA, C., NEGRI, E., DECARLI, A., FASOLI, M. & CISLAGHI, C. (1990). Cancer mortality in Italy: an overview of age-specific and age standardised trends from 1955 to 1984. *Tumori*, **76**, 87.

LONGNECKER, M.P., ORZA, M.J., ADAMS, M.E., VIOQUE, J. & CHALMERS, T.C. (1990). A meta-analysis of alchoholic beverage consumption in relation to risk of colorectal cancer. *Cancer Causes & Control*, **1**, 59.

MCMICHAEL, A.J., POTTER, J.D. & HETZEL, B.S. (1979). Time trends in colo-rectal cancer mortality in relation to food and alcohol consumption: United States, United Kingdom, Australia and New Zealand. *Int. J. Epidemiol.*, **8**, 295.

MCMICHAEL, A.J. & POTTER, J.D. (1980). Reproduction, endogenous and exogenous sex hormones, and colon cancer: a review and hypothesis. *J. Natl Cancer Inst.*, **65**, 1201.

MCMICHAEL, A.J. & GILES, G.G. (1988). Cancer in migrants to Australia: extending the descriptive epidemiological data. *Cancer Res.*, **48**, 751.

MILLER, A.B. (1982). Risk factors from geographic epidemiology for gastrointestinal cancer. *Cancer*, **50**, 2533.

MUIR, C., WATERHOUSE, J., MACK, T., POWELL, J. & WHELAN, S. (eds.) (1987). *Cancer Incidence in Five Continents*, Volume V. International Agency for Research on Cancer, Lyon.

NAIR, P.P. (1984). Diet, nutrition intake, and metabolism in populations at high and low risk for colon cancer. Introduction: correlates of diet, nutrient intake, and metabolism in relation to colon cancer. *Am. J. Clin. Nutr.*, **40**, 880.

NATIONAL HEALTH STATISTICS CENTRE (1982). *Mortality and Demographic Data 1979*. Department of Health, Wellington: New Zealand.

OSMOND, C., GARDNER, M.J., ACHESON, E.D. & ADELSTEIN, A.M. (1983). *Trends in cancer mortality 1951–1980: analysis by period of birth and death*. London: Her Majesty's Stationary Office, Series DH1 no.1.

ROSE, R.J. (1967). *Medical Statistics Report. Part 1-Mortality and Demographic Data 1965*. National Health Statistics Centre: Department of Health, Wellington: New Zealand.

SHIMIZU, H., MACK, T.M., ROSS, R.K. & HENDERSON, B.E. (1987). Cancer of the gastrointestinal tract among Japanese and white immigrants in Los Angeles county. *J. Natl Cancer Inst.*, **78**, 223.

SIMPSON, A. (1986). Variations in operation rates in New Zealand. *N.Z. Med. J.*, **99**, 798.

SMITH, A.H., PEARCE, N.E. & JOSEPH, J.G. (1985). Major colorectal cancer aetiological hypotheses do not explain mortality trends among Maori and non-Maori New Zealanders. *Int J. Epidemiol.*, **14**, 79.

TAYLOR, R., HENDERSON, B., LEVY, S., KOLONEL, L. & LEWIS, N. (1985). *Cancer in Pacific Island Countries*. Information Document No. 53, Noumea: South Pacific Commission, New Caledonia.

WELLS, J.E. (1989). Alcohol disorders: 1976–1985. In *Contemporary Health Issues*. National Health Statistics Centre, Department of Health, Wellington: New Zealand.

WILLETT, W. (1989). The search for the causes of breast and colon cancer. *Nature*, **338**, 389.

ZARIDZE, D.G. (1983). Environmental etiology of large-bowel cancer. *J. Natl Cancer Inst.*, **70**, 389.